# Long-term survivor of relapsed MFH on the thigh treated with autologous formalin-fixed tumor vaccine (AFTV) combined with limb-sparing surgery and radiotherapy

**DOI:** 10.1186/1477-7819-9-96

**Published:** 2011-08-24

**Authors:** Takeshi Todoroki, Tadashi Kondo, Shinji Sugahara, Yukio Morishita, Kensaku Mori, Tadao Ohno

**Affiliations:** 1 Department of Surgery, Tsukuba Central Hospital, Ushiku-shi 300-121, Japan; 2 Department of Surgery, Tsukuba University, Tsukuba-Shi 305-8575, Japan; 3 Department of Therapeutic Radiology, Tsukuba University, Tsukuba-Shi 305-8575, Japan; 4 Clinical Pathology, Tsukuba University, Tsukuba-Shi 305-8575, Japan; 5 Diagnostic Radiology of Tsukuba University, Tsukuba-Shi 305-8575, Japan; 6 Cell-Medicine Inc. Tsukuba-Shi 305-0047, Japan

## Abstract

Malignant fibrous histiocytoma (MFH) is an aggressive spindle cell cancer of soft-tissue sarcoma type in the elderly, mostly affecting the extremities. Lesions > 5 cm, positive margins, and local recurrence are significant poor prognostic indicators. The strongest predictor for distant metastasis was tumor size (> 5 cm), and for overall survival, presence of local recurrence. Limb-sparing extensive tumor resection is preferred to achieve negative surgical margins. However, in some circumstances, amputation is inevitable. Recent studies demonstrated that adjuvant radiotherapy for microscopically positive surgical margins significantly improved local control and disease-free survival rates. Therefore, effective therapeutic strategies against locally relapsed high grade MFH are required to prevent distant metastasis and to achieve long-term disease-free survival. Here, we report local relapse of high grade MFH treated by successive application of autologous formalin-fixed tumor vaccination (AFTV) with limb-sparing surgery and postoperative radiotherapy. The patient is alive and well, disease-free and with no functional impairment, more than five years after treatment.

## Background

Malignant fibrous histiocytoma (MFH) is the most common soft-tissue sarcoma. When located in a limb, MFH is currently treated with limb-sparing surgery followed by > 65 Gy external beam radiotherapy [1,2]. For patients with locally recurrent large tumors (> 5 cm in diameter) on the thigh, limb-sparing surgery with adequate clearance margins is desirable but not always attainable. Additionally, large tumors often result in distant metastasis [1], for which there is currently no effective chemotherapy [2]. Patients thus face amputation for complete tumor removal; so improved treatment options are urgently required for locally recurrent high-grade MFH. Here, we report application of autologous formalin-fixed tumor vaccination (AFTV) combined with limb-sparing surgery and followed by anticipatory 74 Gy external electron beam radiotherapy in an elderly diabetic patient with locally recurrent MFH after primary limb-sparing surgery. The vaccine was prepared from both the recurrent and the primary tumor after limb-sparing surgery [3].

## Case report

**Figure 1:**
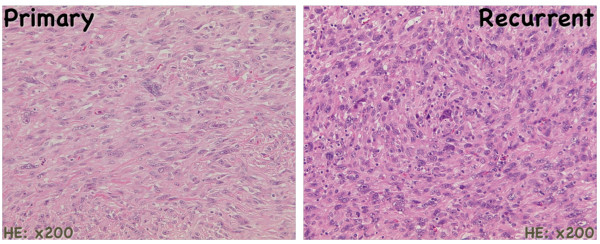
**microscopic photograps (HE: X200)**. Primary (left) and Recurrent (right) tumors are consisted of proliferative fibroblastoid cells, bizarre nucleated cells with high mitotic activity and partially necrotic areas.

**Figure 2:**
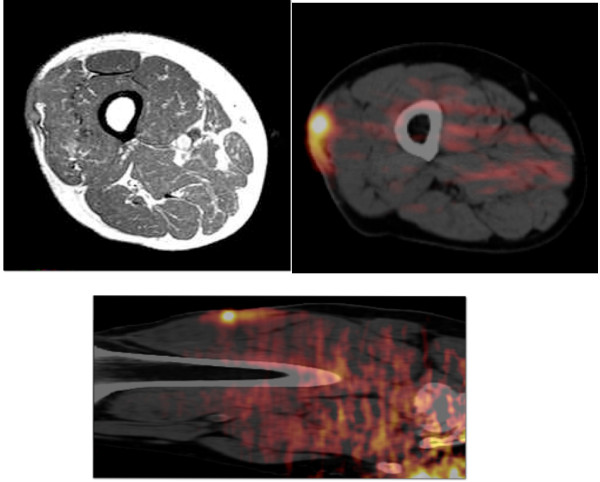
**MRI & PET-CT**. A: MRI image demonstrates a small recurrent tumor on the vertical surgical margin at the fascia lata of the right thigh. B: PET-CT mages in coronary (1) and sagittal (2) sections of the right thigh and hot spots indicate the recurrent tumor.

**Figure 3:**
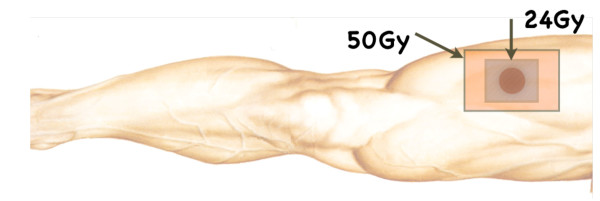
**Post-operative radiation fields**. Post-operative radiation fields in shrinking field technique.

**Figure 4:**
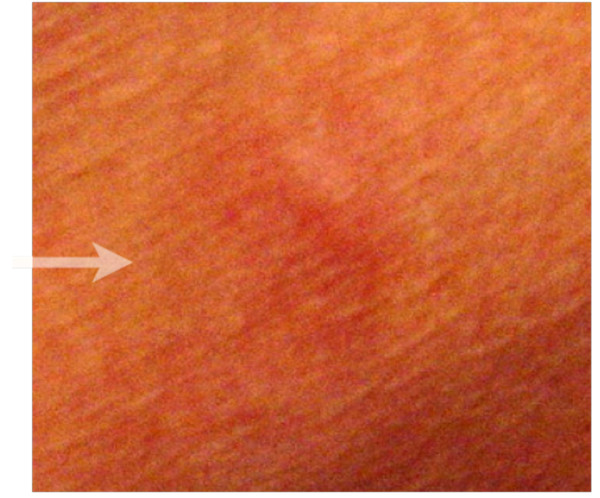
**Autologuos tumor specific delayed type hypersensitivity test (DTH)**. White arrow indicates pseudo-positive reaction.

**Figure 5:**
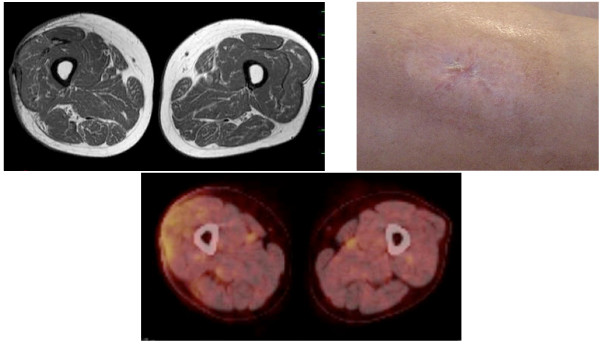
**Coronary sections of MRI and PET-CT, and Photograph on the right thigh**. No tumor exist 30 months after treatments on these images.

**Figure 6:**
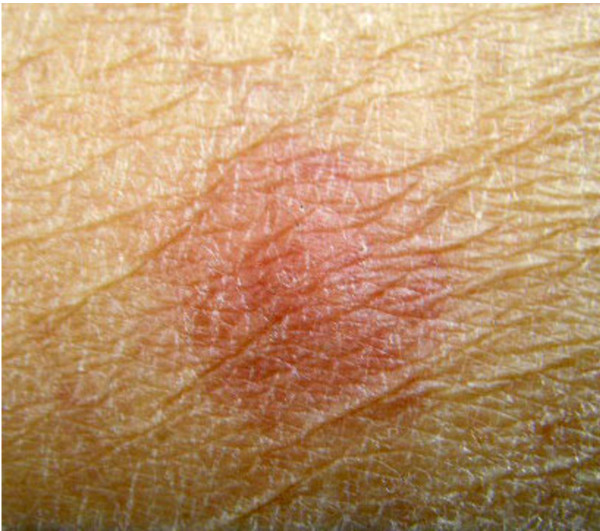
**Positive DTH test**. Positive DTH test performed around 4 years after completion of AFTV.

A 72-year old man presented with a painful ulcerative tumor (4.5 cm) at the location of a scar on the right thigh, where a mass 7.5 cm in diameter had been resected at a different clinic three months earlier. No pathological information was available at the time, but because of the painful infected lesion, we promptly removed the tumor, which had cloudy exudates at the ulcerative surface. The patient had suffered > 5 years from poorly controlled diabetes mellitus (DM) with blood sugar at 272 mg/dl and an elevated HbA1C of 9.4%, but no ketone bodies in the urine. Other laboratory tests revealed signs of slight inflammation, including a WBC of 6,540/mm^3 ^and a CRP of 0.35 mg/dl. We removed the tumor with 1.5 cm horizontal margins, preserving the fascia lata. DM was treated orally. Pathological examination revealed that the mass was a malignant fibrous histiocytoma (MFH) composed of strongly proliferating fibroblastic and histiocytic tumor cells with bizarre nuclei, abundant mitotic activity, a pleomorphic pattern and necrotic areas. These findings were identical in the primary extirpated tumor (7.5 cm) reported in the meantime by the previous clinic (Figure 1). This corresponds to the UICC classification of high grade (G3-deep) MFH, stage III [4]. Because pathology showed that the vertical surgical margin was positive, magnetic resonance imaging (MRI) and positron emission computed tomography (PET-CT) was undertaken 5 weeks after surgery. This revealed small mass at the vertical surgical margin, but no metastatic lesions (Figure 2). Beginning 6 weeks after the second tumor removal, electron beam adjuvant radiotherapy using a shrinking field technique was used to deliver a total dose of 74 Gy in fractions of 2.0 Gy over 5 consecutive days per week (Figure 3). Initial 6 MV X-ray was irradiated by using lateral opposed field with 15-degree wedge filter and 5 mm bolus to surgical scar and tumor bed up to 50 Gy/25 fractions/5 weeks. After that, 24 Gy/12 fractions/2.4 weeks of irradiation was delivered to surgical scar with 6 MeV electron beam as a boost. Paraffin-embedded primary tumor and the formalin-fixed recurrent tumor were injected intradermally three times every two weeks as AFTV. Delayed-type hypersensitivity (DTH) testing both before the first injection and 48 hours later was negative. DTH two weeks after the final AFTV injection was judged pseudo-positive (Figure 4). Immune parameters after treatment were normalized (Additional File 1). The treatment course was uneventful; there were no adverse effects. Around 5 years after the primary limb-sparing surgery the patient remains well with no evidence of metastasis or local recurrence (Figure 5) and the third DTH test performed around 4 years after AFTV was apparently positive (Figure 6).

## Discussion and conclusions

Combining limb-sparing surgery and postoperative radiotherapy with > 65 Gy is standard treatment for patients with high grade large MFH [1]. Treatment of local recurrence of such tumors would also require systemic adjuvant therapy to prevent distant metastasis. However, no effective chemotherapeutic regimen exists. Here, we applied AFTV from the start of postoperative radiotherapy instead of chemotherapy, in consideration of the patient's uncontrolled DM. Details of the AFTV have already been reported [5]. We used a mixture of both paraffin-embedded primary tumor and formalin-fixed secondary tumor as the vaccine. In this way, we aimed to include the original tumor antigens in the primary tumor as well as any possible alterations of the expressed tumor antigens in the progressive metastatic tumor cells. The objective was to eradicate the microscopically observed tumor cells remaining after surgery and irradiation. Hafner et al. reported that decreased immune surveillance might play a role in the development of MFH, based on a significant increase in its incidence in a large series of renal transplant patients (156 per 100,000) [6]. This was another reason to attempt AFTV rather than chemotherapy. Even in the present time, there is no clinically appropriate estimating system of antitumor cellular immune reactivity for the particular patient. Since DTH testing is commonly used to measure specific antitumor cellular immune reactivity we used it to evaluate antitumor cellular immune status in the different timing as just before, complete, and late after AFTV. The reactivity has strengthened from pseudo and true positive during 4 years after AFTV in the reported case. Radiation therapy of 74 Gy by using shrinking field technique has very efficiently controlled locally relapse high grade MFH. As conclusion, results for the case reported here suggest that AFTV in combination with limb-sparing surgery and adjuvant radiotherapy might extend survival of patients with locally advanced high grade MFH of extremities with enhancing specific antitumor immune reactivity.

## Consent

Written consent was obtained from the patient for publication of this case report and accompanying images. A copy of the written consent is available for review by the Editor-in-Chief of this journal.

## Competing interests

The authors declare that they have no competing interests.

## Authors' contributions

TK carried out postoperative wound care. SS participated in the postoperative radiotherapy. MT participated in the pathological studies. KM participated in the radiological imaging studies. TO participated in preparing AFTV treatment design and coordination. All authors read and approved the final manuscript.

## Supplementary Material

Additional file 1 **Table1**. Immune Parameters 2 wk after completion of AFTV. Click here for file
