# Supplementary material for: Long-term survivor of relapsed MFH on the thigh treated with autologous formalin-fixed tumor vaccine (AFTV) combined with limb-sparing surgery and radiotherapy
Source: World J Surg Oncol. 2011 Aug 24;9:96. doi: 10.1186/1477-7819-9-96 (PMC3176478; doi:10.1186/1477-7819-9-96)
Supplement: Additional file 1 — Table1. Immune Parameters 2 wk after completion of AFTV. [file 1477-7819-9-96-S1.DOC]

**Table 1. Immune Parameters 2 wk after completion of AFTV**

| CD3 | T-cell | 63.0% | Normal range |
| --- | --- | --- | --- |
| CD4 | Helper T | 45.2% | Normal range |
| CD8 | Suppressor T | 19.9% | Low |
| CD4/CD8 ratio | 2.27 |  | Normal range |
| CD56 | NK cell | 22.5% | Normal range |
| CD57 | NK cell | 28.7% | Normal range |
| CD25 | Activated T | 16.2% | Normal range |
